# Financial accessibility of healthcare: characteristics of people who refrain from healthcare due to costs over the period 2016–2024, a repeated cross-sectional study

**DOI:** 10.1186/s12913-026-14672-2

**Published:** 2026-05-11

**Authors:** S. Huijgen, M. A. Meijer, A. E. M. Brabers, J. D. de Jong

**Affiliations:** 1 Nivel (Netherlands Institute for Health Services Research), Utrecht, The Netherlands; 2 Care and Public Health Research Institute, Maastricht University, Maastricht, The Netherlands

**Keywords:** Refraining from care, Income, Financial situation, Patient compliance, Netherlands, Questionnaire research

## Abstract

**Background:**

Affordability is an important dimension of access to healthcare. Out-of-pocket payments could lead to refraining from care. Current increased costs of living and care may lead to certain groups of vulnerable people, like low-income groups, being more likely to refrain from care due to costs. This study aims to assess the characteristics of people who refrain from healthcare due to costs over the period 2016–2024.

**Methods:**

Questionnaires were sent out to samples of 1,500 panel members of the Nivel Dutch Health Care Consumer Panel in the period 2016–2024 (response 41%-56%). The samples were representative of the Dutch adult population regarding age and gender. Multivariate logistic regression analyses were performed to identify characteristics associated with refraining from care due to costs. Refraining from care due to costs was operationalised as refraining from (1) seeing a doctor, (2) a medical examination, treatment or post-treatment and/or (3) medication.

**Results:**

Percentages of refraining from care due to costs ranged from 7% to 16%. A higher income, better financial situation, better self-reported health, being older, and having no migration background were related to lower odds of refraining from care due to costs. When income and financial situation are taken together in the analysis, only financial situation plays a role in refraining from care due to costs. In addition, we found that from 2021 onwards, income is no longer associated with refraining from care due to costs. The association of financial situation with refraining from care due to costs increases over the years 2019–2024.

**Conclusions:**

Financial situation seems to play a more important role in refraining from care due to costs than net monthly income. It is important that, when countering refraining from care due to costs, attention is paid to the whole group of people with a poorer financial situation and not only to those with low incomes.

**Supplementary Information:**

The online version contains supplementary material available at 10.1186/s12913-026-14672-2.

## Background

Access to healthcare is an important element in creating a high-quality healthcare system. Access to care can be viewed as a general concept that is “*a measure of the “fit” between characteristics of providers and health services and characteristics and expectations of clients*,* and includes five reasonably distinct dimensions: availability*,* accessibility*,* accommodation*,* affordability and acceptability*” [1]. This study will focus on the dimension of affordability, which is the pricing of healthcare services in relation to people’s ability and willingness to pay for care [1]. People might experience difficulties with the affordability of care, for example, due to out-of-pocket payments. If care is unaffordable, this may lead to people refraining from healthcare due to costs [2]. Refraining from care is defined as someone who does not seek healthcare, despite the perceived need for it [3]. It is an important indicator for inequalities in access to healthcare [4].

Refraining from care due to costs can be both desirable and undesirable. Desired refraining from care includes refraining from non-necessary care and does not have negative consequences for society and the person themselves. Undesired refraining from care involves avoiding necessary care. This can cause symptoms to worsen, resulting in possible complications. It may result in additional health damage for a person, a higher risk of (longer) hospitalisation, poorer self-reported health, and a lower quality of life [5–7]. Due to the higher risk of (longer) hospitalisation and complications or co-morbidities, refraining from necessary healthcare might increase healthcare costs for society [6, 8].

Research has shown that several characteristics are associated with refraining from care due to costs. First, several personal characteristics of people who refrain from care due to costs are identified in the literature. Examples of such characteristics are gender, age, civil status, and living situation [9–16]. In addition, studies identify characteristics related to people’s care and health. Self-reported health, smoking, physical health, mental health and chronic conditions were identified as relevant characteristics [3, 11, 12, 15–17]. Lastly, several characteristics related to the finances of people were identified. Examples of financial characteristics are financial leeway, income, educational level, type of work, deductibles for care, and employment status [3, 9, 11–19].

In the Netherlands, being the focus of this study, affordability of care is an important pillar of the healthcare system [20]. All citizens are obliged to obtain basic health insurance (see box 1.1 for more information). For this basic health insurance, a monthly premium must be paid. A compulsory deductible must be paid for care covered by this basic health insurance. This compulsory deductible does not apply for, for example, general practitioners. Furthermore, a co-payment applies to certain care included in the basic health insurance, for example, for a specific treatment or medicine. In addition, some care, which is not included in the basic health insurance, must be fully paid for by oneself unless people have supplementary insurance that covers these costs. The (additional) costs can cause people to refrain from healthcare when they cannot afford the out-of-pocket payments. In the Netherlands, several studies have been conducted to determine the extent of refraining from healthcare due to costs. The reported percentages range between 3% and 21% [3, 6, 16, 25–28]. This variety of percentages is due to different research populations and/or definitions of refraining from healthcare used by these studies.

**Box 1.1:** Funding of the Dutch healthcare system

Citizens’ mandatory health insurance contributions largely fund the Dutch healthcare system [21]. In addition to the nominal premium for the basic insurance, the healthcare system is financed through income taxes. As the Dutch healthcare system is based on managed competition between health insurers, the premium for the basic health insurance is determined by health insurers. All basic insurance packages cover the same healthcare. Having basic insurance is compulsory for everyone who lives or works in the Netherlands. The basic health insurance includes, among others, general practitioner care, hospital care, home nursing care, pharmaceutical care, and mental healthcare. In the period 2016–2025, the first €385 of care covered by basic insurance must be paid out of pocket. This is called the compulsory deductible. This compulsory deductible does not apply to general practitioner care
consultations, maternity care, and home nursing care [21]. Compared to other countries, the Netherlands has relatively low out-of-pocket payments [22]. In addition to the compulsory deductible, an additional voluntary deductible of up to €500 can be chosen. The compulsory and voluntary deductible must be paid before the insurer will reimburse other healthcare costs. The aim of this deductible is to control healthcare expenditures through shifting funds and raising patient awareness of costs [23]. For citizens with lower incomes, there are possibilities to apply for healthcare benefits. In 2024, 4.82 million people in the Netherlands received healthcare benefits, 27% of the population [24]. Healthcare that is not covered by the basic health insurance can be insured via supplementary insurance, which is not obligatory [21].

In the Netherlands, people are currently faced with increased costs of living due to inflation and increased healthcare premiums [27, 29, 30]. There are concerns that these higher costs will lead to more people refraining from care [27]. Due to this development, this study will focus on the extent of refraining from care due to costs and the role of background characteristics of people who refrain from care over the period 2016–2024. Based on available literature that identified groups of people who are more likely to refrain from care due to costs, we expect that rising costs will cause low-income groups and younger citizens to refrain from healthcare more often [3, 6, 11, 12, 15–19, 31]. By identifying relevant characteristics, appropriate support can be provided to specific groups. To our knowledge, this is the first study to examine the association between background characteristics and refraining from care due to costs over a longer period of time, thereby being able to provide insight into the degree to which the associations between these characteristics and refraining from care due to costs have changed over time. We answer the following research questions:

- Q1: What is the development of refraining from care due to costs in the Netherland in the period 2016–2024?
- Q2: What are the background characteristics of people who refrain from care due to costs in the period 2016–2024?
- Q3: Have the background characteristics of people who refrain from care due to costs changed over the period 2016–2024?

## Methods

### Setting

Data obtained within the Dutch Health Care Consumer Panel were used for this study. The panel is managed by Nivel (Netherlands Institute for Health Services Research) and aims to assess Dutch people’s expectations and experiences with healthcare and their opinions and knowledge about the Dutch healthcare system [32]. In 2024, the panel consisted of 10,500 people from the general Dutch population aged 18 years and older. Information about several background characteristics of the panel members, such as their age and educational level, is known. Members of the panel have agreed to answer questionnaires on a regular basis. Membership to the panel is by invitation only. Members of the Dutch Health Care Consumer Panel are informed of the purpose, scope, method, and use of the panel upon joining it. Based on this information, participants can give their permission to take part in the panel. A written informed consent, which, since 2020, can also be digital, is obtained at the time of the registration of a new member. Panel members can stop their membership at any time without having to give a reason. By returning their questionnaire, the respondents were considered to have consented to participate. Panel members receive points for completing questionnaires. If panel members collect enough points, they receive a gift card. Pseudonymized data were analysed and processed in accordance with the privacy policy of the Dutch Health Care Consumer Panel. The panel complies with the General Data Protection Regulation (GDPR) [32]. According to Dutch legislation, approval by a medical ethics committee is not obligatory for conducting research through a panel [33]. The Review Committee of the Amsterdam UMC has confirmed that research conducted through the Dutch Health Care Consumer Panel is not subject to approval by a medical ethics committee (2024.1150).

### Questionnaire

Questionnaires were sent out to samples of 1,500 members of the Dutch Health Care Consumer Panel in November in the years 2016–2024. Each year a new sample of respondents was drawn from the total group of 10,500 panel members. All the samples were representative of the Dutch adult population with regard to age and gender. The samples do not consist of the same panel members, although there was some overlap due to selection criteria (see 2.4). Among the questions included were those about refraining from care due to costs. The questionnaire could be filled in online or by post, depending on the personal preferences of the panel members. To increase the response, reminders were sent out to respondents who had not yet completed the questionnaire. The lowest response rate was 41% in 2016, and the highest response rate was 56% in 2021.

### Measures

#### Refraining from care due to costs

To determine refraining from care due to costs, the OECD formulated three questions [34]. We translated these questions to Dutch for our study. These OECD questions were also asked and published by various countries to enable comparison. These questions focus on refraining from (1) seeing a doctor, (2) a medical examination, treatment, or post-treatment, and (3) picking up a prescription for medication or skipping doses of medicine, all due to costs. All three questions could be answered with No or Yes (recoded into 0 = No, 1 = Yes), whereby the respondents answering No consist both of those who sought care when they needed it, and those who did not need care. Based on these three questions, the variable ‘refraining from at least one form of care due to costs’ was created. In case a respondent answered ‘yes’ to one or more of these questions, the respondent scored ‘Yes’ on the new variable. In the remainder of this article the term ‘refraining from care due to costs’ is used.

#### Background variables

Supplementary file 1 presents the background characteristics included in this study. The background characteristics were selected after a literature study. The goal of this literature study was to identify which background characteristics of people are known to be associated with refraining from care due to costs. In this literature study, we managed to identify different background characteristics [3, 9, 11, 12, 15]. We then examined which demographic characteristics identified in the literature were represented in our Dutch Health Care Consumer Panel. If there was data for this particular demographic characteristic, that characteristic was selected for the analysis.

### Statistical analysis

At first, descriptive analyses were performed to describe respondents’ background characteristics, such as age and gender.

To determine the percentage of people who refrained from care due to costs in the Netherlands in the period 2016–2024 (Q1), descriptive analyses were performed. A logistic regression analysis was performed to determine whether refraining from care due to costs increases or decreases over time. The logistic regression analysis took into account any possible clustering effects resulting from the overlap in respondents. Since the response in each year was not completely representative of the general Dutch adult population regarding age and gender, weight factors were applied. Six categories were distinguished, based on three age categories (18–39 years, 40–64 years, and 65 years and older) and two categories for gender (men and women). Weight factors varied between 0.60 and 4.19.

To determine what background characteristics are associated with refraining from care (Q2), logistic regression models were performed. Firstly, we studied the association of each individual background characteristic and refraining from care due to costs. After this, based on a content-driven approach, the statistically significant characteristics were clustered into three themes: personal characteristics, care/health characteristics and financial characteristics, and we examined the associations between the clustered characteristics and refraining from care due to costs (for more information see [35]). As the final step, the statistically significant background characteristics from the clustered analyses were combined in a multiple regression analysis. The logistic regression models took into account any possible clustering effects resulting from the overlap in respondents.

To answer Q3, interaction models were used to determine whether the significant background characteristics in refraining from care due to costs have changed over time. Regression coefficients for the association of year were estimated for each subgroup by including interactions between year and the different groups. To correct for the influence of other background characteristics that may also be associated with refraining from care due to costs, these characteristics were added in the interaction model. The background characteristics that emerged as significant from the multiple logistic regression analyses, were used for the interactions. To correct for multiple testing, the Bonferroni correction was applied.

Due to the overlap in respondents in het different samples, 2,569 respondents completed the questionnaire once and 3,804 completed the questionnaire more than once. All analyses were performed using Stata, version 16.1. Unless otherwise specified, a significance level of 5% (*P* = 0.05) was maintained in the analyses.

## Results

### Description of the respondents

The distribution of the included background characteristics of people in the descriptive analyses, such as age, gender, and net monthly income, and the percentage of people refraining from care due to costs, both per year and for all years taken together, can be found in Supplementary file 1. Among other things, Supplementary file 1 shows that across all years, 51% of respondents were female. In addition, half (50%) reported their health as good and 51% were 40 to 64 years old.

### Refraining from care due to costs over the years

**Fig. 1:**
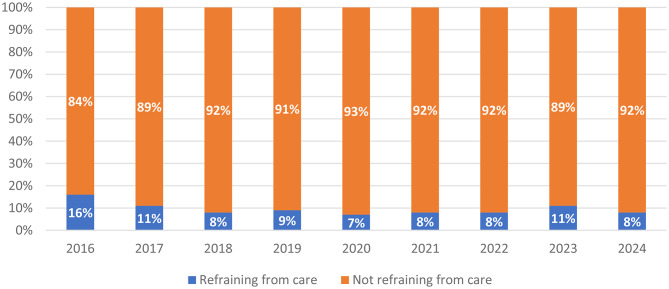
Percentage of respondents who indicated they refrained care due to costs (*N* = 601–837, weighed)

Figure 1 shows that the percentage of people that refrain from care due to costs ranges between 7% (2020) and 16% (2016). Using a logistic regression analysis, we found that refraining from care due to costs has significantly decreased over time (see Supplementary file 2 for the output). This indicates that the percentage of people who indicated that they refrained from care due to costs has decreased between 2016 and 2024. This decline was mainly in the first years, and the rate has since 2018 been fluctuating between 7 and 11% for several years.

### Background characteristics related to refraining from care due to costs

Net monthly household income, financial situation, social position (e.g. paid work or retirement), self-reported health, age and migration background are associated in the univariate and cluster analyses with refraining from care due to costs (see Additional file 3 and 4). On this basis, these characteristics were included in the multivariate analyses. We built three multivariate regression models (see Table 1). The first model (model 0) included all variables that were found to be associated with refraining from care due to costs. This showed that when both income and financial situation are included, only financial situation is statistically significant. As data on financial situation are only available for five years, we decided to also create a model including only net monthly income (model 1), as well as a model including only financial situation (model 2). The other variables are available in all years.

In *model 1* for net monthly income, it was observed that people with an income of 1750 up to 2700 euros (OR = 0.68) and > 2700 euros (OR = 0.44) are less likely to refrain from care than people with an income < 1750 euros. For self-reported health, people with an excellent/very good self-reported health (OR = 0.41) and a good self-reported health (OR = 0.54) are less likely to refrain from care than people with a moderate/bad self-reported health. Also for age, it was found that people of 65 years and older (OR = 0.22) are less likely to refrain from care than people between 18 and 39 years. Regarding migration background, people without a migration background (OR = 0.53) are less likely to refrain from care than people with a migration background.

For *model 2* regarding financial situation, people who can make ends meet exactly (OR = 0.54) and people who can save a little money/save a lot money (OR = 0.20) are less likely to refrain from care than people who need to go into debt/are addressing savings. Regarding self-reported health, the group with an excellent/very good self-reported health (OR = 0.52) and good self-reported health (OR = 0.58) are less likely to refrain from care than people with moderate/bad self-reported health. For age, people of 65 years and older are less likely (OR = 0.30) to refrain from care than people of 18 up to 39 years. Lastly, for migration background, people without a migration background (OR = 0.60) are less likely to refrain from care due to costs than people with a migration background.

**Table 1:** Results of multivariate logistic regression analysis with included background characteristics^1^

		Refraining from at least one form of care due to costs (model 0, inclusion of both income and financial situation, *N* = 3.075^2^)		Refraining from at least one form of care due to costs (model 1, inclusion of income, *N* = 5.503^2^)		Refraining from at least one form of care due to costs (model 2, inclusion of financial situation, *N* = 3.207^2^)	
		Odds Ratio (95%CI)	P-values	Odds Ratio (95%CI)	P-value	Odds ratio (95%CI)	P-value
Net monthly household income	Less than 1750 euros	Ref		Ref			
	1750 up to 2700 euros	1.16 (0.77–1.73)	0.49	0.68 (0.52–0.91)	0.01*		
	More than 2700 euros	1.10 (0.70–1.72)	0.68	0.44 (0.33–0.58)	0.00*		
Financial situation	I need to go into debt/I am addressing savings	Ref				Ref	
	I can make ends meet exactly	0.58 (0.39–0.87)	0.01*			0.54 (0.37–0.80)	0.00*
	I save a little money/I save a lot of money	0.21 (0.14–0.33)	0.00*			0.20 (0.14–0.30)	0.00*
Social position	Housewife/male	Ref		Ref		Ref	
	Going to school/studying	1.40 (0.49–4.04)	0.53	1.21 (0.50–2.91)	0.67	1.53 (0.53–4.39)	0.43
	Paid work	0.62 (0.25–1.53)	0.30	0.73 (0.35–1.52)	0.40	0.70 (0.29–1.70)	0.43
	Unemployed	0.56 (0.18–1.72)	0.31	1.34 (0.60–2.97)	0.47	0.58 (0.19–1.76)	0.33
	Incapacitated	0.67 (0.25–1.81)	0.43	1.15 (0.53–2.53)	0.72	0.80 (0.30–2.10)	0.65
	Retirement	0.92 (0.33–2.56)	0.87	0.91 (0.38–2.18)	0.84	1.02 (0.36–2.87)	0.97
	Other	0.84 (0.25–2.80)	0.77	1.05 (0.40–2.76)	0.92	0.86 (0.26–2.87)	0.81
Self-reported health	Moderate/Bad	Ref		Ref		Ref	
	Excellent/Very good	0.51 (0.33–0.80)	0.00*	0.41 (0.29–0.59)	0.00*	0.52 (0.34–0.81)	0.00*
	Good	0.57 (0.40–0.82)	0.00*	0.54 (0.41–0.72)	0.00*	0.58 (0.40–0.82)	0.00*
Age	18 up to 39 years	Ref		Ref		Ref	
	40 up to 64 years	0.84 (0.55–1.29)	0.43	0.74 (0.53–1.02)	0.06	0.86 (0.56–1.30)	0.47
	65 years and older	0.30 (0.12–0.78)	0.01*	0.22 (0.10–0.48)	0.00*	0.30 (0.12–0.76)	0.01*
Migration background	Western/non-Western migration background	Ref		Ref		Ref	
	No migration background	0.64 (0.38–1.07)	0.09	0.53 (0.37–0.77)	0.00*	0.60 (0.36–0.99)	0.04*

^1^An asterisk following the p-value indicates a significant p-value

^2^N is the number of observations included in the model, due to missing values the numbers differ from the numbers mentioned in the Methods section

### Share of background characteristics in refraining from care over time

To determine the possible change in association with refraining from care due to costs, the statistical significance of interactions between the background characteristics and the years 2016–2024 was examined (see Additional file 5).

For net monthly household income, the association with refraining from care due to costs has decreased over time. From 2021, groups with a higher net monthly income and groups with a lower net monthly income no longer significantly differ in their likelihood of refraining from care due to costs. This means that from 2021 onwards, income no longer has an (significant) influence on refraining from care due to costs.

For financial situation, the statistical significance of interactions was determined between the different background characteristics and the years 2019–2024. This was decided, as this characteristic was only measured in these years. For financial situation, the association with refraining from care due to costs between the categories ‘I save a little money/I save a lot of money’ and ‘I need to go into debt/I am tapping into my savings’ is significant in the period 2021–2024 and the association increases. This means that from 2021 onwards, financial situation has an (significant) influence on refraining from care due to costs.

For the characteristics self-reported health, age, and migration background there are no clear patterns in the associations on refraining from care due to costs over time.

## Discussion

The aim of this study was to assess the extent of refraining from care due to costs in the Netherlands over the period 2016–2024. In addition, characteristics of people who refrain from care due to costs were studied. This study has been conducted because, to our knowledge, no study has yet been conducted into the association between background characteristics and refraining from care due to costs over time. This study showed that the percentage of people in the Netherlands who indicated that they refrained from care due to costs ranged between 7% (2020) and 16% (2016). We also found differences between groups in refraining from care due to costs. Having a better self-reported health, being older, having no migration background, having a higher net monthly income, and a better financial situation are associated with a lower likelihood of refraining from care due to costs. Furthermore, when income and financial situation are taken together in the analyses, only financial situation plays a role in refraining from care due to costs. In addition, it seems that financial situation plays a bigger role in refraining from care due to costs than income.

The percentage of people refraining from care due to costs was higher in 2016 than in other years. As 2016 was the first year we measured refraining from care, we do not have an explanation for this finding. The percentages of refraining from care due to costs are a bit lower in this study than the percentages found in other research performed in the Netherlands. For example, the Dutch Patient Federation found that in 2023 21% of people in the Netherlands refrained from care due to costs [36] and Salampessy, Portrait [37] found that 14% of people in the Netherlands refrained from care due to the deductible. Differences in observed percentages are probably the result of differences in the definition of refraining from care due to costs and the different target groups used in the studies. For example, the study of the Dutch Patient Federation was focused on patients, while we focused on the general population. In addition, the percentages found in this study are lower than the reported percentages in other countries. In 2020, 14% refrained from a medical examination or treatment in Switzerland [38]. In the Netherlands this percentage is lower, as 7% refrained from at least one form of care due to costs in 2020. The height of out-of-pocket payments could potentially explain the different percentages of refraining from care due to costs. In the Netherlands, the out-of-pocket payments are relatively low compared to other countries. In 2019, out-of-pocket payments in the Netherlands were, according to the international definition, 10.6% of current healthcare expenditure. By comparison, the rate in Switzerland was 25.3% of the current healthcare expenditure, in Germany the rate was 12.7% of the current healthcare expenditure, and in Belgium the rate was 18.2% of the current healthcare expenditure [39]. High out-of-pocket payments may lead to people having difficulty with the affordability of care, leading people to refrain from care, which could explain the higher percentage in Switzerland [3, 40].

We found that having a better self-reported health, being older, having no migration background, having a higher net monthly income, and having a better financial situation are associated with lower odds of refraining from care due to costs. These results correspond with existing literature. For the characteristic self-reported health, Litwin and Sapir [41] provide two explanations for why people with a poorer self-reported health are more likely to refrain from care due to costs. First, people with poorer health need more care and therefore have a greater opportunity to refrain from care due to costs. Secondly, people who need several treatments tend to prioritize the ones deemed the most important and refrain from the other treatments. However, regarding the relationship between refraining from care due to costs and self-reported health it can be reasoned that it works both ways. Poorer self-reported health might lead to more refraining from care, and refraining from care might lead to poorer self-reported health. With regards to age, Salampessy, Portrait [3] showed that older age is a protective factor for refraining from care due to costs. Older individuals are possibly more aware of possible adverse effects of refraining from care due to costs because of their age and previous experience with healthcare use [3]. Besides this, younger people might have more competing financial demands, such as housing, than older people [16]. Lastly, literature shows that groups with a migration background may experience socioeconomic disadvantages and may have encountered negative experiences and interactions with the healthcare system, which makes them more likely to refrain from care [9, 16].

We found that financial situation appears to play a greater role in refraining from care due to costs than net monthly income. This is consistent with research by Salampessy, Portrait [3], however, the authors gave no explanation for this. A possible explanation is that what someone has left at the end of the month might be more important than income. Our study shows that over the years, the share of income in refraining from care due to costs decreased and the share of financial situation increased in general. Financial situation therefore seems to play a bigger role in refraining from care due to costs than income. A possible explanation for the fact that in the last years financial situation is more important than income, is that people in the Netherlands have been facing rising energy prices and high levels of inflation [29, 42]. Low-income households receive various forms of financial assistance, such as healthcare, housing, and energy benefits [43, 44]. Higher-income households do not receive these benefits. As a result, there are people with a middle income who struggle to make ends meet [45]. Because of their poorer financial situation, they may choose to refrain from care. This possibly makes what people have left at the end of the month more important than net monthly income. It is, therefore, important that, when addressing refraining from healthcare due to costs, attention is paid to the entire group of people in a poorer financial situation, and not just to those with low incomes. Reducing the compulsory deductible that people have to pay out-of-pocket for healthcare can ensure that people in a poorer financial situation, as well as those with lower incomes, are less inclined to refrain from care due to costs, as the costs when using healthcare are lower. A disadvantage might be that premiums will be higher when deductibles are reduced, as such increasing costs for all insured.

### Recommendations for further research

Ideally, future research should focus on the development of refraining from care due to costs using a longitudinal study design. With this study design, several years of data would be available from the same sample of respondents instead of data from different samples used in our study. It makes it possible to understand the cause-and-effect relationship of the different background characteristics and refraining from care due to costs.

In this study, it was not possible to distinguish between (1) desired refraining of care, whereby people did not use care because they did not need it, and (2) undesired refraining of care, whereby people did not use care even though they needed it. The percentages of refraining from care due to costs reported in this study include both desired and undesired refraining from care due to costs. Since desired refraining from care due to costs is not a problem for the patient and/or society, it may be that the percentages in this study overestimate the problem. In order to make statements on the severity of refraining from care due to costs, future research should focus on undesired refraining from care due to costs specifically. This undesired refraining from care due to costs can be more problematic for some groups than others, for example due to differences in health status.

Lastly, future research into refraining from care due to costs should take into account people’s overall financial situation and not just their income.

### Strengths and limitations

A strength of this study is the inclusion of data on refraining from care due to costs from nine different years. In this way, this study not only provides insight into the extent of refraining from care due to costs and relevant background characteristics of people who refrain from care at one point in time but also into the development over time. To our knowledge, this is the first study to focus on the background characteristics of people who refrain from care due to costs over time. The large sample size of the study and the possibility to fill out the questionnaire both online and on paper are other strengths of the study.

A limitation of this study is that in examining the percentage of people who refrained from care due to costs, we were not able to distinguish between desired and undesired refraining from care due to costs. Refraining from care due to costs may be more problematic for some groups than others. People may have indicated they refrained from care due to costs, but their symptoms went away on their own. This is a form of refraining from care where there is no harm to the patient and/or society. It is therefore not possible to make statements on the severity of refraining from care due to costs. For future research it is recommended to include the perceived consequences of refraining from care due to costs, to study whether these consequences differ for different groups. Another limitation is that the net monthly household income has not been adjusted for household size, as we have no data on this. A large number of family members means that the income has to be shared among more people than in the case of a small number of family members, which influences the financial situation of people. However, we were unable to correct for this.

Lastly, a limitation might be that we used self-reported data from questionnaires. The analyses in this study were conducted using data from questionnaires. In addition, citizens who are illiterate or who do not speak Dutch, or homeless people are not able to participate in questionnaires. As a result, the most vulnerable groups, who may be more likely to refrain from care due to costs, could not be included in the study.

## Conclusion

Our results show that better self-reported health, being older, having no migration background, having a higher net monthly income, and having a better financial situation are associated with lower odds of refraining from care due to costs. Financial situation appears to play a greater role in refraining from care due to costs than net monthly income. This means that what someone has left at the end of the month is more important in refraining from care due to costs than net monthly income. It is, therefore, important that, when addressing refraining from care due to costs through policy, attention is not only paid to net monthly income but to the entire group of people in a poorer financial situation. 

## Supplementary Information

Below is the link to the electronic supplementary material.

Supplementary Material 1: Appendix A- Descriptive statistics respondents

Supplementary Material 2: Appendix B- Time trend analysis

Supplementary Material 3: Appendix C- Univariate analysis

Supplementary Material 4: Appendix D- Cluster analysis

Supplementary Material 5: Appendix E- Interaction models

## Data Availability

A minimal, anonymised dataset is available upon reasonable request from prof. Judith de Jong J.dejong@nivel.nl, project leader of the Dutch Health Care Consumer Panel. The Panel has a program committee, which supervises processing the data of the panel and decides about the use of the data. This program committee consists of representatives of the Dutch Ministry of Health, Welfare and Sport, the Health Care Inspectorate, Zorgverzekeraars Nederland (Association of Health Care Insurers in the Netherlands), the National Health Care Institute, the Federation of Patients, the Dutch Healthcare Authority, and the Dutch Consumers Association. All research conducted within the panel has to be approved by this program committee. The committee assesses whether a specific research fits within the aim of the Dutch Health Care Consumer Panel, which is to strengthen the position of the health care user.

## Abbreviation

GP: General Practitioner
