# Supplementary material for: Financial accessibility of healthcare: characteristics of people who refrain from healthcare due to costs over the period 2016–2024, a repeated cross-sectional study
Source: BMC Health Serv Res. 2026 May 11;26:913. doi: 10.1186/s12913-026-14672-2 (PMC13340322; doi:10.1186/s12913-026-14672-2)
Supplement: Supplementary file 1 — Supplementary Material 1: Appendix A- Descriptive statistics respondents [file 12913_2026_14672_MOESM1_ESM.pdf]

	2016 (N=616)	2017 (N=668)	2018 (N=645)	2019 (N=664)	2020 (N=701)	2021 (N=837)	2022 (N=737)	2023 (N=723)	2024 (N=782)	Total (N=6373)
**Gender**										
Male	300 (49%)	344 (52%)	319 (49%)	339 (51%)	325 (46%)	395 (47%)	323 (44%)	374 (52%)	398 (51%)	3.117 (49%)
Female	316 (51%)	324 (49%)	326 (51%)	325 (49%)	376 (54%)	442 (53%)	414 (56%)	349 (48%)	384 (49%)	3.256 (51%)
**Age**										
18 up to 39 year	116 (19%)	133 (20%)	107 (17%)	90 (14%)	73 (10%)	174 (21%)	113 (15%)	162 (22%)	195 (25%)	1.163 (18%)
40 up to 64 year	296 (48%)	363 (54%)	319 (49%)	398 (60%)	408 (58%)	412 (49%)	383 (52%)	327 (45%)	339 (43%)	3.245 (51%)
65 years and older	204 (33%)	172 (26%)	219 (34%)	176 (27%)	220 (31%)	251 (30%)	241 (33%)	234 (32%)	248 (32%)	1.965 (31%)
**Social position**										
Going to school/studying	26 (4%)	28 (4%)	18 (3%)	22 (3%)	21 (3%)	37 (4%)	24 (3%)	28 (4%)	21 (3%)	225 (4%)
Paid work	298 (49%)	352 (53%)	331 (52%)	383 (58%)	379 (55%)	465 (56%)	402 (55%)	367 (55%)	362 (55%)	3.339 (54%)
Unemployed	20 (3%)	46 (7%)	34 (5%)	28 (4%)	35 (5%)	28 (3%)	25 (3%)	15 (2%)	26 (4%)	257 (4%)
Incapacitated	30 (5%)	45 (7%)	40 (6%)	40 (6%)	32 (5%)	42 (5%)	50 (7%)	34 (5%)	40 (6%)	353 (6%)
Housewife/male	26 (4%)	20 (3%)	23 (4%)	23 (3%)	24 (4%)	24 (3%)	29 (4%)	16 (2%)	15 (2%)	200 (3%)
Retirement	201 (33%)	153 (23%)	180 (28%)	140 (21%)	175 (26%)	211 (25%)	187 (26%)	188 (28%)	181 (27%)	1.616 (26%)
Other, namely	11 (2%)	21 (3%)	15 (2%)	26 (4%)	20 (3%)	26 (3%)	11 (2%)	18 (3%)	14 (2%)	162 (3%)
**Self-reported health**										
Excellent/Very good	203 (33%)	164 (27%)	195 (30%)	203 (33%)	225 (34%)	260 (36%)	238 (35%)	219 (33%)	222 (30%)	1.929 (32%)
Good	328 (54%)	343 (56%)	337 (53%)	303 (49%)	339 (51%)	336 (46%)	302 (44%)	324 (48%)	362 (49%)	2.974 (50%)
Moderate/Poor	82 (13%)	107 (17%)	109 (17%)	117 (19%)	105 (16%)	135 (18%)	144 (21%)	128 (19%)	149 (20%)	1.076 (18%)
**Financial situation**										
I need to go into debt/I am addressing savings				69 (11%)		51 (7%)	73 (11%)	59 (9%)	51 (7%)	303 (9%)
I can make ends meet exactly				139 (22%)		161 (22%)	166 (24%)	131 (20%)	135 (19%)	732 (21%)
I save a little money/I save a lot of money				415 (67%)		513 (71%)	439 (65%)	478 (72%)	539 (74%)	2.384 (70%)
**Net monthly household income**										
Less than 1750 euros	187 (32%)	179 (28%)	193 (31%)	146 (23%)	184 (28%)	115 (16%)	106 (16%)	90 (14%)	65 (9%)	1.265 (22%)

1750 up to 2700 euros	179 (31%)	215 (33%)	196 (32%)	192 (30%)	201 (31%)	208 (30%)	175 (27%)	170 (26%)	178 (25%)	1.714 (29%)
More than 2700 euros	216 (37%)	249 (39%)	233 (37%)	301 (47%)	269 (41%)	381 (54%)	368 (57%)	390 (60%)	461 (65%)	2.868 (49%)
**Single vs. multi-person household**										
Single household	160 (26%)	155 (23%)	160 (25%)	145 (22%)	190 (28%)	233 (28%)	185 (25%)	181 (27%)	190 (29%)	1.599 (26%)
Multi-person household	452 (74%)	510 (77%)	479 (75%)	519 (78%)	491 (72%)	601 (72%)	549 (75%)	487 (73%)	470 (71%)	4.558 (74%)
**Educational level**										
Low (up to lower vocational education)	101 (17%)	93 (14%)	118 (19%)	80 (13%)	106 (16%)	79 (10%)	78 (11%)	75 (11%)	81 (11%)	811 (13%)
Middle	318 (53%)	317 (49%)	289 (46%)	288 (45%)	315 (47%)	362 (44%)	323 (45%)	302 (44%)	304 (40%)	2.818 (46%)
High (university of applied sciences, university)	179 (30%)	237 (37%)	226 (36%)	270 (42%)	248 (37%)	382 (46%)	319 (44%)	314 (45%)	368 (49%)	2.543 (41%)
**Marital status**										
Married	354 (58%)	412 (62%)	366 (57%)	416 (63%)	402 (59%)	491 (59%)	449 (61%)	405 (61%)	394 (60%)	3.689 (60%)
Divorced	71 (12%)	57 (9%)	62 (10%)	69 (10%)	88 (13%)	85 (10%)	50 (7%)	54 (8%)	58 (9%)	594 (10%)
Widow/widower	57 (9%)	41 (6%)	51 (8%)	30 (5%)	44 (6%)	43 (5%)	54 (7%)	48 (7%)	40 (6%)	408 (7%)
Never been married	128 (21%)	158 (24%)	159 (25%)	146 (22%)	147 (22%)	211 (25%)	179 (24%)	156 (24%)	164 (25%)	1.448 (24%)
**Number of people living on income**										
One person (only myself)	188 (31%)	195 (30%)	193 (31%)	166 (26%)	188 (28%)	204 (28%)	153 (27%)	158 (31%)	160 (30%)	1.605 (29%)
Multiple people (including myself)	419 (69%)	446 (70%)	426 (69%)	473 (74%)	478 (72%)	514 (72%)	416 (73%)	359 (69%)	373 (70%)	3.904 (71%)
**Chronic condition**										
No chronic condition	359 (59%)	435 (67%)	405 (64%)	445 (68%)	449 (67%)	582 (71%)	492 (68%)	490 (70%)	536 (70%)	4.193 (67%)
Chronic condition	250 (41%)	219 (33%)	226 (36%)	205 (32%)	223 (33%)	243 (29%)	234 (32%)	206 (30%)	225 (30%)	2.031 (33%)
**Migration background**										
No migration background	585 (95%)	614 (92%)	607 (94%)	600 (93%)	635 (93%)	778 (93%)	686 (94%)	659 (94%)	615 (91%)	5.779 (93%)
Western/non-Western migration background	29 (5%)	53 (8%)	37 (6%)	42 (7%)	51 (7%)	58 (7%)	47 (6%)	43 (6%)	58 (9%)	418 (7%)
**Children living at home**										
No children living at home	426 (70%)	457 (69%)	451 (71%)	444 (67%)	420 (62%)	539 (65%)	457 (62%)	420 (63%)	428 (65%)	4.042 (66%)
Children living at home	186	208	188	220	261	295	277	247	232	2.114

	(30%)	(31%)	(29%)	(33%)	(38%)	(35%)	(38%)	(37%)	(35%)	(34%)
**Healthcare use**										
None				54 (9%)	77 (12%)	58 (8%)	151 (22%)	61 (9%)	72 (10%)	473 (12%)
(Very) little				464 (74%)	489 (73%)	553 (75%)	427 (63%)	493 (73%)	516 (71%)	2.942 (72%)
(Very) much				106 (17%)	101 (15%)	122 (17%)	105 (15%)	119 (18%)	142 (19%)	695 (17%)
**Deductible incurred**										
Yes				268 (43%)		326 (45%)	347 (51%)	322 (48%)	368 (50%)	1.631 (48%)
No				313 (50%)		352 (48%)	283 (42%)	301 (45%)	303 (42%)	1.552 (45%)
I do not know				42 (7%)		52 (7%)	50 (7%)	48 (7%)	59 (8%)	251 (7%)
**Contact GP**										
0/1 time		256 (41%)		272 (43%)		325 (45%)		279 (41%)		1.132 (43%)
2/3/4 times		295 (47%)		285 (45%)		314 (43%)		313 (46%)		1.207 (45%)
5 times or more		79 (13%)		71 (11%)		87 (12%)		85 (13%)		322 (12%)
**Refraining from at least one form of care due to costs**										
Yes	79 (16%)	71 (11%)	49 (8%)	48 (9%)	40 (7%)	65 (8%)	60 (8%)	73 (11%)	60 (8%)	
No	522 (84%)	589 (89%)	594 (92%)	593 (91%)	659 (93%)	772 (92%)	677 (92%)	648 (89%)	722 (92%)	
