# Supplementary material for: Financial accessibility of healthcare: characteristics of people who refrain from healthcare due to costs over the period 2016–2024, a repeated cross-sectional study
Source: BMC Health Serv Res. 2026 May 11;26:913. doi: 10.1186/s12913-026-14672-2 (PMC13340322; doi:10.1186/s12913-026-14672-2)
Supplement: Supplementary file 2 — Supplementary Material 2: Appendix B- Time trend analysis [file 12913_2026_14672_MOESM2_ESM.pdf]

Table 1: Output logistic regression analysis time trend (N=6,321)

```
Logistic regression                             Number of obs     =      6,321
                                                Wald chi2(11)     =    1947.37
Log pseudolikelihood = -1811.8659               Prob > chi2       =     0.0000

                             (Std. Err. adjusted for 3,890 clusters in copanr)
```

kosten1	Coef.	Robust Std. Err.	z	P>|z|	[95% Conf.	Interval]
jaar						
2016	-.8050601	.272887	-2.95	0.003	-1.339909	-.2702115
2017	-1.069889	.2580987	-4.15	0.000	-1.575753	-.5640246
2018	-1.398076	.2797784	-5.00	0.000	-1.946432	-.8497201
2019	-1.432295	.2829336	-5.06	0.000	-1.986834	-.8777551
2020	-1.69157	.2836207	-5.96	0.000	-2.247457	-1.135684
2021	-1.40993	.2640047	-5.34	0.000	-1.927369	-.8924899
2022	-1.340913	.2765812	-4.85	0.000	-1.883003	-.7988244
2023	-1.129866	.2673316	-4.23	0.000	-1.653826	-.6059056
2024	-1.453908	.2754588	-5.28	0.000	-1.993797	-.9140188
lft	-.0211917	.0029799	-7.11	0.000	-.0270321	-.0153513
geslacht	.0706379	.1085484	0.65	0.515	-.1421131	.2833889

```
Contrasts of predictive margins                 Number of obs     =      6,321
Model VCE    : Robust

Expression   : Pr(kosten1), predict()
```

	df	chi2	P>chi2
jaar (linear)	1	7.37	0.0066

	Contrast	Delta-method Std. Err.	[95% Conf.	Interval]
jaar (linear)	-.0102552	.0037763	-.0176567	-.0028537
