# Supplementary material for: Financial accessibility of healthcare: characteristics of people who refrain from healthcare due to costs over the period 2016–2024, a repeated cross-sectional study
Source: BMC Health Serv Res. 2026 May 11;26:913. doi: 10.1186/s12913-026-14672-2 (PMC13340322; doi:10.1186/s12913-026-14672-2)
Supplement: Supplementary file 3 — Supplementary Material 3: Appendix C- Univariate analysis [file 12913_2026_14672_MOESM3_ESM.pdf]

		Refraining from at least one form of care due to costs (N=2658-6321)	
		Odds ratio (95%CI)	P-value
Contact GP	0/1 times	0.46 (0.30-0.70)	0.00*
	2/3/4 times	0.76 (0.52-1.11)	0.16
	5 times or more	Ref	
Own risk incurred	Yes	1.13 (0.70-1.84)	0.62
	No	0.71 (0.43-1.18)	0.18
	I do not know	Ref	
Self-reported health	Excellent/Very good	0.35 (0.27-0.47)	0.00*
	Good	0.46 (0.36-0.58)	0.00*
	Moderate/Bad	Ref	
Having a chronic condition	No chronic condition	0.87 (0.69-1.08)	0.21
	Chronic condition	Ref	
Healthcare use	None	Ref	
	(Very) little	1.57 (1.01-2.45)	0.046*
	(Very) much	2.44 (1.47-4.03)	0.00*
Financial situation	I need to go into debt/I am tapping into my savings	Ref	
	I can make ends meet exactly	0.47 (0.33-0.67)	0.00*
	I save a little money/I save a lot of money	0.17 (0.12-0.24)	0.00*
Net monthly income	Less than 1750 euros	Ref	
	1750 up to 2700 euros	0.57 (0.44-0.74)	0.00*
	More than 2700 euros	0.42 (0.33-0.54)	0.00*
Having children living at home	No children living at home	0.82 (0.65-1.02)	0.08
	Children living at home	Ref	
Gender	Male	Ref	
	Female	1.10 (0.90-1.36)	0.35
Migration background	No migration background	0.46 (0.34-0.63)	0.00*
	Western/non-Western migration background	Ref	
Age	18 up to 39 year	Ref	
	40 up to 64 year	0.86 (0.67-1.12)	0.27
	65 year and older	0.34 (0.25-0.47)	0.00*
Marital status	Married	1.72 (1.07-2.78)	0.03*
	Divorced	2.69 (1.56-4.63)	0.00*
	Widow/widower	Ref	
	Never been married	2.09 (1.27-3.43)	0.00*
Social position	Going to school/studying	1.24 (0.58-2.66)	0.59
	Paid work	0.67 (0.35-1.28)	0.22
	Unemployed	1.77 (0.85-3.69)	0.13
	Incapacitated	1.97 (0.97-3.99)	0.06

		Refraining from at least one form of care due to costs (N=2658-6321)	
		Odds ratio (95%CI)	P-value
	Housewife/male	Ref	
	Retirement	0.37 (0.19-0.72)	0.00*
	Other	1.14 (0.47-2.77)	0.77
Educational level	Low	Ref	
	Middle	0.92 (0.67-1.28)	0.63
	High (university of applied sciences, university)	0.80 (0.58-1.11)	0.18
Single vs. multi-person household	Single person household	Ref	
	Multiple-person household	1.06 (0.84-1.34)	0.61
