# Supplementary material for: Financial accessibility of healthcare: characteristics of people who refrain from healthcare due to costs over the period 2016–2024, a repeated cross-sectional study
Source: BMC Health Serv Res. 2026 May 11;26:913. doi: 10.1186/s12913-026-14672-2 (PMC13340322; doi:10.1186/s12913-026-14672-2)
Supplement: Supplementary file 4 — Supplementary Material 4: Appendix D- Cluster analysis [file 12913_2026_14672_MOESM4_ESM.pdf]

Table 1 odds ratios and p-values for cluster analyses personal background characteristics

		Refraining from at least one form of care due to costs (N=6.050)	
		Odds Ratio (95%CI)	P-value
Children living at home	No children living at home	1.13 (0.85-1.50)	0.40
	Children living at home	Ref	
Marital status	Married	0.91 (0.51-1.65)	0.77
	Divorced	1.55 (0.87-2.76)	0.14
	Widow/widower	Ref	
	Never been married	0.91 (0.50-1.63)	0.74
Migration background	No migration background	0.47 (0.34-0.65)	0.00*
	Western/non-Western migration background	Ref	
Age	18 up to 39 year	Ref	
	40 up to 64 year	0.86 (0.63-1.19)	0.37
	65 years and older	0.31 (0.20-0.48)	0.00*
Gender	Male	Ref	
	Female	1.11 (0.89-1.39)	0.34
Single vs. multi-person household	Single household	Ref	
	Multi-person household	1.09 (0.78-1.54)	0.61

**Significant odds ratio*

Table 2 odds ratios and p-values for cluster analyses (health)care characteristics model 1 and 2[1]

		Refraining from at least one form of care due to costs (model 1, N=1.948)		Refraining from at least one form of care due to costs (model 2, N=5831)	
		Odds Ratio (95%CI)	P-value	Odds ratio (95%CI)	P-value
Healthcare use	Non	Ref			
	(Very) little	1.56 (0.70-3.44)	0.28		
	(Very) much	1.52 (0.60-3.82)	0.37		
Chronic condition	No chronic condition	0.98 (0.66-1.46)	0.93	1.10 (0.86-1.41)	0.43
	Chronic condition	Ref		Ref	
Self-reported health	Excellent/Very good	0.41 (0.23-0.74)	0.00*	0.34 (0.25-0.46)	0.00*
	Good	0.45 (0.28-0.71)	0.00*	0.45 (0.35-0.57)	0.00*
	Moderate/Bad	Ref		Ref	
Contact GP	0/1 time	0.74 (0.40-1.37)	0.33		
	2/3/4 times	1.10 (0.66-1.82)	0.72		
	5 times or more	Ref			
Deductible incurred	Yes	0.96 (0.48-1.92)	0.91		
	No	0.95 (0.46-1.96)	0.89		
	I do not know	Ref			

**Significant odds ratio*

[1]The variables healthcare use, contact GP, and deductible incurred were not questioned in all years, which is why a second model was created for this cluster. This model only included the characteristics that were questioned in every year.

Table 3 odds ratio and p-values for cluster analyses financial characteristics model 1 and 2

		Refraining from at least one form of care due to costs (model 1, N=3.039)		Refraining from at least one form of care due to costs (model 2, N=5.498)	
		Odds Ratio (95%CI)	P-value	Odds Ratio (95%CI)	P-value
Net monthly household income	Less than 1750 euros	Ref		Ref	
	1750 up to 2700 euros	1.17 (0.78-1.75)	0.45	0.65 (0.49-0.86)	0.00*
	More than 2700 euros	1.03 (0.65-1.64)	0.89	0.38 (0.28-0.52)	0.00*
Financial situation	I need to go into debt/I am addressing savings	Ref			
	I can make ends meet exactly	0.57 (0.38-0.86)	0.01*		
	I save a little money/I save a lot of money	0.20 (0.13-0.30)	0.00*		
Educational level	Low	Ref		Ref	
	Middle	1.10 (0.66-1.84)	0.71	0.99 (0.70-1.41)	0.97
	High (university of applied sciences, university)	1.17 (0.68-2.00)	0.56	1.05 (0.72-1.55)	0.79
Social position	Going to school/studying	1.66 (0.60-4.56)	0.33	1.48 (0.65-3.37)	0.35
	Paid work	0.70 (0.30-1.66)	0.42	0.83 (0.42-1.67)	0.61
	Unemployed	0.66 (0.22-1.99)	0.46	1.65 (0.77-3.54)	0.20
	Incapacitated	0.91 (0.34-2.42)	0.85	1.57 (0.73-3.35)	0.25
	Housewife/male	Ref		Ref	
	Retirement	0.43 (0.17-1.06)	0.07	0.36 (0.17-0.73)	0.01*
	Other	0.89 (0.27-2.96)	0.85	1.22 (0.48-3.12)	0.67

**Significant odds ratio*
