# Supplementary material for: Financial accessibility of healthcare: characteristics of people who refrain from healthcare due to costs over the period 2016–2024, a repeated cross-sectional study
Source: BMC Health Serv Res. 2026 May 11;26:913. doi: 10.1186/s12913-026-14672-2 (PMC13340322; doi:10.1186/s12913-026-14672-2)
Supplement: Supplementary file 5 — Supplementary Material 5: Appendix E- Interaction models [file 12913_2026_14672_MOESM5_ESM.pdf]

Table 1 interaction models with contrast values and p-values

Year	Interaction	Contrast (b)	p-value (Bonferroni correction)
Self-reported health (p< 0.00185)			
2016	Good # Excellent/Very good	0.35	0.23
2016	Moderate/Poor # Excellent/Very good	0.88	0.02
2016	Moderate/Poor # Good	0.53	0.12
2017	Good # Excellent/Very good	0.72	0.06
2017	Moderate/Poor # Excellent/Very good	1.33	0.00
2017	Moderate/poor # Good	0.61	0.06
2018	Good/Excellent # Very good	0.58	0.23
2018	Moderate/Poor # Excellent/Very good	1.90	0.00
2018	Moderate/Poor # Good	1.32	0.00
2019	Good # Excellent/Very good	0.09	0.83
2019	Moderate/Poor # Excellent/Very good	0.84	0.06
2019	Moderate/Poor # Good	0.75	0.05
2020	Good # Excellent/Very good	-0.40	0.33
2020	Moderate/Poor # Excellent/Very good	0.30	0.53
2020	Moderate/Poor # Good	0.69	0.14
2021	Good # Excellent/Very good	0.45	0.25
2021	Moderate/Poor # Excellent/Very good	1.04	0.02
2021	Moderate/Poor # Good	0.59	0.11
2022	Good # Excellent/Very good	0.29	0.47
2022	Moderate/Poor # Excellent/Very good	1.40	0.00
2022	Moderate/Poor # Good	1.11	0.00
2023	Good # Excellent/Very good	0.22	0.51
2023	Moderate/Poor # Excellent/Very good	1.04	0.01
2023	Moderate/Poor # Good	0.82	0.02
2024	Good # Excellent/Very	0.06	0.89

	good		
2024	Moderate/Poor # Excellent/Very good	0.20	0.66
2024	Moderate/Poor # Good	0.15	0.73
Net monthly income (p< 0.00185)			
2016	>2700 euros # <1750 euros	-.099	0.00
2016	1750-2700 euros # <1750 euros	-0.58	0.06
2016	>2700 euros # 1750-2700 euros	-0.41	0.24
2017	1750-2700 euros # <1750 euros	-1.18	0.00
2017	>2700 euros # <1750 euros	-1.13	0.00
2017	>2700 euros # 1750-2700 euros	0.06	0.89
2018	1750-2700 euros # <1750 euros	-0.25	0.45
2018	>2700 euros # <1750 euros	-1.81	0.00
2018	>2700 euros # 1750-2700 euros	-1.56	0.00
2019	1750-2700 euros # <1750 euros	0.55	0.16
2019	>2700 euros # <1750 euros	-1.03	0.03
2019	>2700 euros # 1750-2700 euros	-1.58	0.00
2020	1750-2700 # <1750 euros	-0.49	0.24
2020	>2700 euros # <1750 euros	-1.81	0.00
2020	>2700 euros # 1750-2700 euros	-1.32	0.02
2021	1750-2700 euros # <1750 euros	-0.62	0.14
2021	>2700 euros # <1750 euros	-1.04	0.01
2021	>2700 euros # 1750-2700 euros	-0.42	0.27
2022	1750-2700 euros # <1750 euros	-0.31	0.46
2022	>2700 euros # <1750 euros	-0.58	0.12
2022	>2700 euros # 1750-	-0.27	0.45

	2700 euros		
2023	1750-2700 euros # <1750 euros	-0.21	0.63
2023	>2700 euros # <1750 euros	-0.43	0.27
2023	>2700 euros # 1750-2700 euros	-0.22	0.52
2024	1750-2700 euros # <1750 euros	-0.53	0.39
2024	>2700 euros # <1750 euros	-0.23	0.67
2024	>2700 euros # 1750-2700 euros	0.30	0.48
Age (p< 0.00185)			
2016	40-64 years # 18-39 years	-0.22	0.45
2016	65 years and older # 18-39 years	-2.83	0.00
2016	65 years and older # 40-64 years	-2.61	0.00
2017	40-64 years # 18-39 years	-0.32	0.34
2017	65 years and older # 18-39 years	-1.51	0.00
2017	65 years and older # 40-64 years	-1.19	0.00
2018	40-64 years # 18-39 years	-0.29	0.45
2018	65 years and older # 18-39 years	-1.85	0.00
2018	65 years and older # 40-64 years	-1.55	0.00
2019	40-64 years # 18-39 years	-1.06	0.01
2019	65 years and older # 18-39 years	-1.42	0.01
2019	65 years and older # 40-64 years	-0.35	0.41
2020	40-64 years # 18-39 years	-0.42	0.40
2020	65 years and older # 18-39 years	-0.89	0.12
2020	65 years and older # 40-64 years	-0.47	0.27
2021	40-64 years # 18-39 years	0.25	0.53

2021	65 years and older # 18-39 years	-0.77	0.13
2021	65 years and older # 40-64 years	-1.03	0.01
2022	40-64 years # 18-39 years	-0.10	0.80
2022	65 years and older # 18-39 years	-0.87	0.06
2022	65 years and older # 40-64 years	-0.77	0.03
2023	40-64 years # 18-39 years	-0.60	0.07
2023	65 years and older # 18-39 years	-1.91	0.00
2023	65 years and older # 40-64 years	-1.31	0.00
2024	40-64 years # 18-39 years	0.24	0.65
2024	65 years and older # 18-39 years	-1.42	0.03
2024	65 years and older # 40-64 years	-1.65	0.00
Migration background (p< 0.0027)			
2016	Western/non-Western migration background # no migration background	1.22	0.004
2017	Western/non-Western migration background # no migration background	1.29	0.00
2018	Western/non-Western migration background # no migration background	0.96	0.02
2019	Western/non-Western migration background # no migration background	0.61	0.26
2020	Western/non-Western migration background # no migration background	-1.20	0.26
2021	Western/non-Western migration background # no migration	0.06	0.91

	background		
2022	Western/non-Western migration background # no migration background	0.35	0.47
2023	Western/non-Western migration background # no migration background	0.82	0.07
2024	Western/non-Western migration background # no migration background	0.41	0.44
Financial situation[1] (p< 0.0028)			
2019	I can make ends meet exactly # I need to go into debt/I am tapping into my savings	0.26	0.59
2019	I save a little money/I save a lot of money # I need to go into debt/I am tapping into my savings	-0.74	0.12
2019	I save a little money/I save a lot of money # I can make ends meet exactly	-1.00	0.00
2021	I can make ends meet exactly # I need to go into debt/I am tapping into my savings	-0.52	0.19
2021	I save a little money/I save a lot of money # I need to go into debt/I am tapping into my savings	-2.16	0.00
2021	I save a little money/I save a lot of money # I can make ends meet exactly	-1.64	0.00
2022	I can make ends meet exactly # I need to go into debt/I am tapping into my savings	-1.09	0.01
2022	I save a little money	-1.49	0.00

[1] This variable was measured in the years 2021-2024. No data is available for this background characteristic for the years 2016-2020

	left/I save a lot of money left # I need to go into debt/I am tapping into my savings		
2022	I save a little money/I save a lot of money # I can make ends meet exactly	-0.40	0.27
2023	I can make ends meet exactly # I need to go into debt/I am tapping into my savings	-0.85	0.03
2023	I save a little money/I save a lot of money # I need to go into debt/I am tapping into my savings	-1.95	0.00
2023	I save a little money/I save a lot money # I can make ends meet exactly	-1.10	0.00
2024	I can make ends meet exactly # I need to go into debt/I am tapping into my savings	-1.01	0.04
2024	I save a little money/I save a lot of money # I need to go into debt/I am tapping into my savings	-1.68	0.00
2024	I save a little money/I save a lot money # I can make ends meet exactly	-0.67	0.10
